# Transcranial Direct Current Stimulation Improves Ipsilateral Selective Muscle Activation in a Frequency Dependent Manner

**DOI:** 10.1371/journal.pone.0122434

**Published:** 2015-03-27

**Authors:** Kazumasa Uehara, James P. Coxon, Winston D. Byblow

**Affiliations:** 1 Movement Neuroscience Laboratory, Department of Sport & Exercise Science, The University of Auckland, Auckland, New Zealand; 2 Centre for Brain Research, The University of Auckland, Auckland, New Zealand; 3 Human Motor Control Laboratory, Division of Human Sciences, Graduate School of Integrated Arts and Sciences, Hiroshima University, Higashi-Hiroshima, Hiroshima, Japan; The University of Western Ontario, CANADA

## Abstract

Failure to suppress antagonist muscles can lead to movement dysfunction, such as the abnormal muscle synergies often seen in the upper limb after stroke. A neurophysiological surrogate of upper limb synergies, the selectivity ratio (SR), can be determined from the ratio of biceps brachii (BB) motor evoked potentials to transcranial magnetic stimulation prior to forearm pronation versus elbow flexion. Surprisingly, cathodal transcranial direct current stimulation (c-TDCS) over *ipsilateral* primary motor cortex (M1) reduces (i.e. improves) the SR in healthy adults, and chronic stroke patients. The ability to suppress antagonist muscles may be exacerbated at high movement rates. The aim of the present study was to investigate whether the selective muscle activation of the biceps brachii (BB) is dependent on altering frequency demands, and whether the c-tDCS improvement of SR is dependent on task frequency. Seventeen healthy participants performed repetitive isometric elbow flexion and forearm pronation at three rates, before and after c-tDCS or sham delivered to ipsilateral left M1. Ipsilateral c-tDCS improved the SR in a frequency dependent manner by selectively suppressing BB antagonist excitability. Our findings confirm that c-tDCS is an effective tool for improving selective muscle activation, and provide novel evidence for its efficacy at rates of movement where it is most likely to benefit task performance.

## Introduction

Selective activation of agonist muscles is necessary for producing skilled and coordinated upper limb movements in daily life. For many reaching and grasping movements, activation of the prime mover (agonist) is associated with simultaneous relaxation of the antagonist [1–3]. Reciprocal inhibition is the neurophysiological mechanism that underlies this functional architecture [4–6]. After central nervous system insult such as after stroke affecting the motor system, selective muscle activation of the paretic limb is often degraded [7–9]. This degradation is noted as impairment and associated with the clinical symptoms of spasticity and hypertonia, and abnormal synergies, such as the flexor synergy involving the stereotypical movement pattern of elbow flexion coupled with forearm pronation and shoulder abduction [7,10].

Transcranial magnetic stimulation (TMS) has been used to investigate the neurophysiological mechanisms of selective muscle recruitment and synergies of the upper limb. Motor evoked potentials (MEPs) elicited in biceps brachii (BB) are suppressed before pronation in healthy individuals but this antagonist suppression is not observed in the most severely impaired stroke patients [11,12]. The relationship between antagonist and agonist function of BB can be expressed as a selectivity ratio (SR) by proportionally representing the size of BB MEPs during forearm pronation to those during elbow flexion [13]. A low SR (e.g., SR = 0.3) supports selective muscle activation. Conversely, a high SR (e.g., S.R. = 0.8) may lead to poor selectivity because BB is not sufficiently suppressed to permit efficient forearm pronation. Overall, stroke patients exhibit higher SRs than healthy controls [11,12] and SR has been shown to correlate with measures of upper limb impairment, and spasticity [12]. Therefore, SR appears to be a neurophysiological surrogate for upper limb synergies.

Both crossed and uncrossed descending pathways innervate the proximal muscles making it likely that pathways originating in the ipsilateral motor cortex influence flexor synergies [14–16]. This assertion is supported by evidence that cathodal tDCS (c-tDCS) applied to ipsilateral M1 reduces the SR measured from the contralateral limb of healthy subjects [17] and in patients with upper limb impairment due to stroke [12]. The ipsilateral primary motor cortex (M1) is thought to contribute to movements of increasing complexity [18,19] and is also modulated by frequency during unilateral rhythmic finger movement [20,21]. In the present study we used c-tDCS to determine if ipsilateral M1 excitability contributes to SR in a frequency dependent manner.

We hypothesized that SR would be worsened at higher rates of contraction where the ability to suppress antagonist activation would be less pronounced. Secondly, given that ipsilateral M1 excitability is modulated by movement frequency [20,21] and c-tDCS of ipsilateral M1 improves SR [12,17], we predicted that SR improvement due to ipsilateral c-tDCS would be more pronounced at higher task frequencies.

## Materials and Methods

### Participants

Twenty healthy adults (mean: 27.0 years, range: 20–34 years, eight female) without history of upper limb neurologic and musculoskeletal disorder participated in this study. Nineteen participants were right-handed (+84.4 ± 16.7, mean ± standard deviation) and one was left-handed (-100), as confirmed by the Edinburgh Handedness Inventory [22]. They were screened for contraindications to TMS and tDCS by a neurologist. The University of Auckland Human Participant Ethics Committee approved this study in accordance with the guidelines established in the Declaration of Helsinki, and written informed consent was obtained from all participants.

### Electromyography recording

Surface electromyography (EMG) was recorded from left BB, left pronator teres (PT) and right first dorsal interosseous (FDI) using disposable Ag/AgCl electrodes (10 mm diameter for left PT and right FDI, Blue Sensor N, Ambu, Denmark, 20 mm diameter for left BB, Red Dot, 3M, United States), following standard skin preparation. For BB and PT, the electrodes were placed over the muscle belly in a bipolar montage. The FDI electrodes were placed in a belly-tendon montage. EMG signals were amplified (CED 1902; Cambridge Electronic Design, Cambridge, United Kingdom), band-pass filtered (10–1000 Hz), sampled at 2 kHz (CED 1401), and stored to computer for offline analysis using Signal software (Signal V4.09).

### Experimental design and protocol

**Fig 1:**
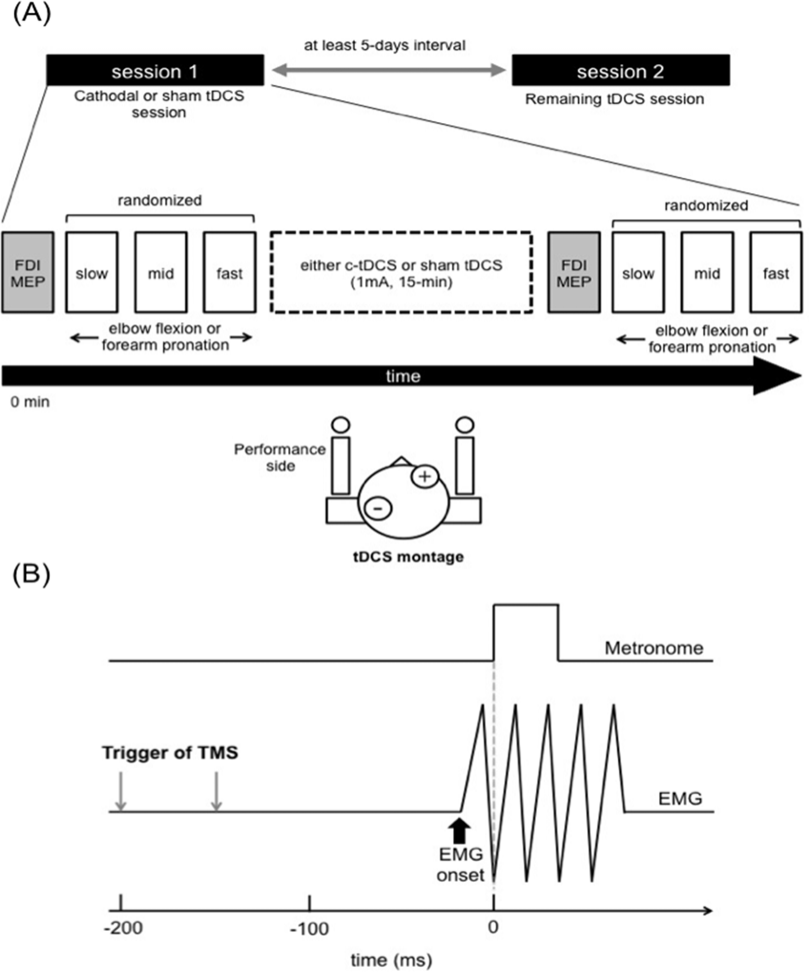
Experiment design, timeline and task. **(A)** Experiment schematic and tDCS montage. All participants completed two experimental sessions, separated by at least 5-days. Before c-tDCS or sham, right FDI MEPs at rest and left BB MEPs prior to muscle contraction were recorded using single-pulse TMS to establish baseline values. c-tDCS (1 mA) or sham was delivered to the M1 ipsilateral to the muscle contraction side for 15 min. After c-tDCS, right FDI MEPs and left BB MEPs measures were repeated. **(B)** An illustration of the motor task and timing of TMS. The participant was required to make discrete left elbow flexion or forearm pronation contractions in time with an auditory stimulus paced at 0.75, 1.0 or 1.25 Hz.

Participants completed two experimental sessions (c-tDCS or sham tDCS) separated by at least 5-days in a randomized double-blind crossover design (Fig. 1A). MEPs were recorded from left BB during three different frequencies of rhythmic elbow flexion or forearm pronation before and after each tDCS session. In order to confirm the efficacy of tDCS over left M1, MEPs in right FDI (i.e., contralateral) were also recorded. The experiment session order was randomized across participants.

### Motor task: elbow flexion and forearm pronation task

Participants were seated on a custom-made chair with their left elbow flexed approximately 90 degrees, the forearm and wrist in a neutral position, and the left hand gripping a vertical bar. The left forearm was restrained using velcro straps to provide resistance when producing brief flexor muscle contraction. The right hand was rested on a custom-made armrest. We asked participants to perform repetitive and brief isometric elbow flexion or forearm pronation, paced at three different frequencies (0.75, 1.0, and 1.25 Hz). Hereafter, we refer to these frequencies as “slow”, “middle”, and “fast”, respectively. Participants were instructed to completely relax their left upper limb between each muscle contraction. Each trial consisted of 50 repetitive muscle contractions at a given frequency. For each task (elbow flexion or forearm pronation), three trials were performed at each frequency both before and after the c-tDCS or sham tDCS intervention (900 per block, 1800 per session). Data collection of each session took around 30 min including around 1 min break between each trial. Visual feedback representing left BB and PT EMG waveforms was displayed on a 24-inch personal computer monitor approximately 1.5 m in front of the participant throughout all experimental sessions. A familiarization trial was performed for each task before the data collection in order to ensure that subjects could perform at each frequency. Single-pulse TMS was delivered over the right M1 while performing each task (see, *TMS* section).

### TMS

Single-pulse TMS was delivered with a figure-of-eight shaped coil (70-mm wing diameter) connected to a Magstim 200 stimulator (Magstim Company, Dyfed, United kingdom). The coil was held tangentially to the scalp with the handle pointing backwards at a 45-degree angle from the sagittal plane, inducing a posterior to anterior current direction within M1 [23,24]. The optimal sites for producing MEPs in left BB and right FDI were determined and marked on a tDCS cap covering the participant’s scalp. Active motor threshold (AMT) for left BB was defined as the minimum intensity that elicited ≥ 100 μV MEPs in four out of eight trials during a weak sustained muscle contraction (around 10% maximum voluntary contraction) of the left BB. The TMS intensity during elbow flexion and forearm pronation was set at 130% of AMT and where necessary, increased to ensure MEPs of sufficient amplitude [11,17]. During the task conditions, single-pulse TMS was delivered over the right M1, either 150 or 200 ms prior to every fifth metronome beat [13] (Fig. 1B). Thirty left BB MEPs were elicited in each task at each frequency (i.e., slow, middle, fast) before and after each tDCS session.

For the measurement of MEPs in right FDI, the TMS intensity was adjusted to elicit an MEP amplitude of 1.0–1.5 mV at rest at the beginning of the experimental session. Single-pulse TMS was delivered over left M1 every 5–7 seconds while participants completely relaxed both upper limbs. Sixteen MEPs were recorded before and immediately after each tDCS session.

### tDCS administration

Cathodal tDCS or sham tDCS was applied over the left M1 (i.e., ipsilateral) using a dedicated tDCS cap (MindCap, Newronika, Italy) and a battery-driven constant current stimulator (HDCstim, Newronika, Italy). For c-tDCS, a current intensity of 1 mA was applied for 15 min through a pair of saline-soaked sponge electrodes (each 25 cm^2^) equating to a maximum current density of 0.04 mA/cm^2^, within safety limits [25]. The electrode montage was such that the cathode was positioned over left M1 and the anode above the right supra orbital area, according to the international 10–20 system (Fig. 1A). For the sham tDCS session, the current was ramped up over 30 seconds and then turned off.

### Data analysis

For the task conditions, root mean squared EMG (rmsEMG) was determined for the 50 ms immediately prior to each TMS stimulus. EMG burst onset in BB or PT was detected when rmsEMG first exceeded 3 standard deviations (SD) above baseline. The TMS-EMG burst onset interval was calculated and traces were discarded from each subject’s data analysis if this interval was < 50 ms or > 250 ms or if left BB prestimulus rmsEMG was > 10μV.

Left BB peak-to-peak MEP amplitudes were obtained from the remaining data. SR was calculated as: SR = mean MEP_BB_ forearm pronation / mean MEP_BB_ elbow flexion, and the tDCS induced effect was determined as: ΔSR = SR_Post_—SR_Pre_. Left BB peak-to-peak MEP amplitudes during elbow flexion and forearm pronation were also analyzed independently.

### Statistical analysis

To confirm the efficacy of c-tDCS, a two-way repeated measures analysis of variance (RM-ANOVA) with factors TIME (pre, post) and STIM (c-tDCS, sham) was performed on right FDI MEP amplitude.

For pre SR and ΔSR, two-way RM-ANOVAs with factors STIM and FREQUENCY (slow, middle, fast) were performed. For pretrigger rmsEMG and TMS-EMG onset interval, three-way RM-ANOVAs with factors STIM, TIME and FREQUENCY were performed. For percentage change in BB MEP amplitude, a three-way RM-ANOVA with factors STIM, TASK (elbow flexion and forearm pronation) and FREQUENCY was performed. Post hoc analyses were employed when necessary and adjusted *P* values are reported using a modified Bonferroni procedure for multiple comparisons [26]. A paired t-test was employed to compare TMS test intensity and AMT of the left BB between sessions. A linear regression analysis with Pearson product-moment correlation coefficient was used to assess the correlation between ΔSR and pre SR as well as between pretrigger rmsEMG and BB MEP. The statistical significance level was set at p < 0.05 for all comparisons and all data are shown as group mean ± standard error (SE).

## Results

None of the participants reported adverse effects from the procedures. Data from 3 participants had to be discarded from the final analysis because they were not able to relax enough between contractions to maintain an average pretrigger rmsEMG below 10μV, and thus there MEP data could not be interpreted.

### Effect of c-tDCS on right FDI MEP amplitude

**Fig 2:**
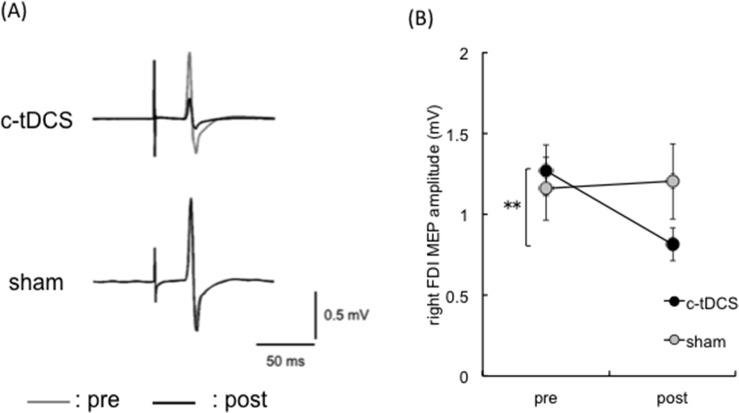
Manipulation check for cathodal transcranial direct current stimulation. **(A)** MEP waveforms (each trace is an average of 16 trials) from right FDI of a representative participant. The gray and black traces indicate pre and post c-tDCS or sham sessions, respectively. **(B)** Mean right FDI MEP amplitude in the c-tDCS (closed circle) and sham (gray circle) sessions (*n = 17*). MEP amplitude decreased after c-tDCS and was unchanged in the sham session. Error bars indicate SE. * p< 0.05; ** p < 0.01.

Typical EMG traces from right FDI of a representative participant are shown in Fig. 2A. There was a main effect of TIME (F_1,16_ = 8.27, p < 0.05), no main effect of STIM (F_1,16_ = 0.53, p = 0.47) and a TIME × STIM interaction (F_1,16_ = 7.91, p < 0.05). Post hoc analyses revealed that right FDI MEP amplitude decreased from pre to post for the c-tDCS session (t_16_ = 3.30, p < 0.01), but not the sham session (t_16_ = -0.54, p = 0.59). This indicates that c-tDCS robustly suppressed the stimulated M1 ipsilateral to the task arm (Fig. 2B).

### c-tDCS effects on left BB SR are frequency dependent

The baseline (i.e., pre) SR values were similar in the c-tDCS and sham sessions (c-tDCS: *Slow* 0.28 ± 0.05, *Middle* 0.32 ± 0.06, *Fast* 0.32 ± 0.06; Sham: *Slow* 0.27 ± 0.05, *Middle* 0.27 ± 0.05, *Fast* 0.28 ± 0.06). There were no main effects of FREQUENCY (F_2,32_ = 0.37, p = 0.69), STIM (F_1,16_ = 0.47, p = 0.50) or FREQUENCY × STIM interaction (F_2,32_ = 0.70, p = 0.50). Thus, baseline SR did not differ for the c-tDCS and sham session at the muscle contraction frequencies tested.

**Fig 3:**
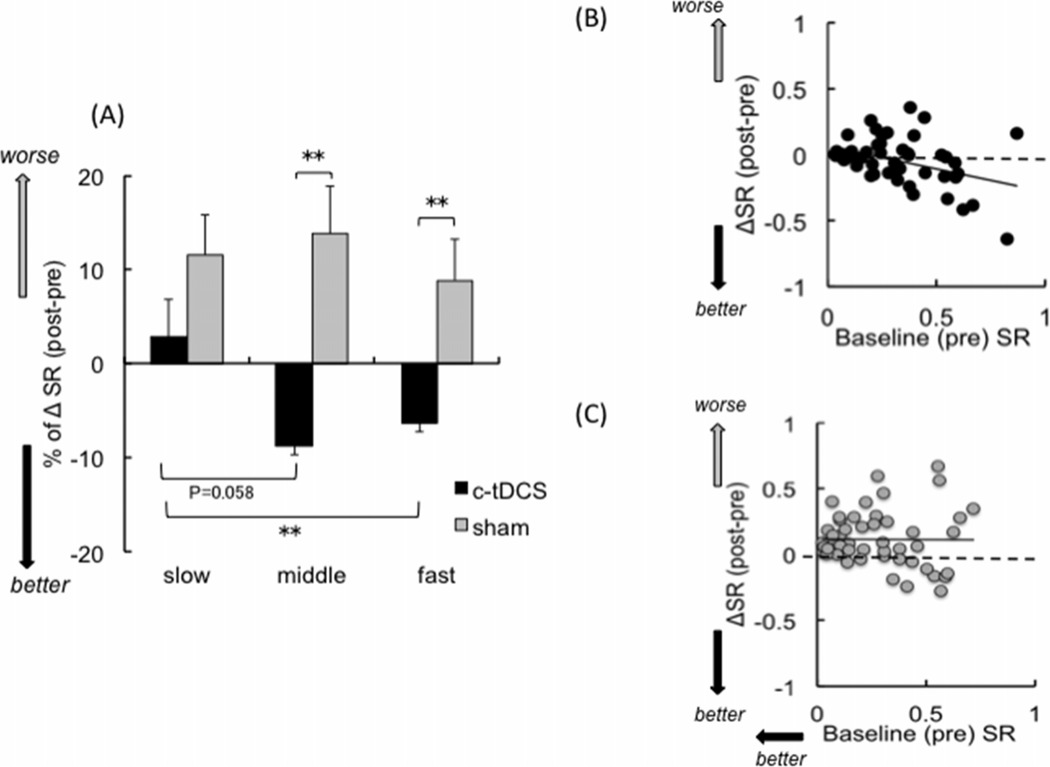
Selectivity ratio analyses. **(A) %**ΔSR *(n = 17)* across the three different frequencies in the c-tDCS (closed bar) and sham (gray bar) sessions. Correlations between ΔSR and baseline SR in the c-tDCS **(B)** and sham **(C)** sessions. Negative numbers indicate improvements of selective muscle activation. Error bars indicate SE. * p < 0.05; **p < 0.01. Slow, middle and fast indicate 0.75, 1.0 and 1.25 Hz, respectively.

The ΔSR (post-pre) values following c-tDCS and sham are shown in Fig. 3A. There was a main effect of STIM (F_1,16_ = 9.41, p < 0.01), no main effect of FREQUENCY (F_2,32_ = 1.85, p = 0.17), and a STIM × FREQUENCY interaction (F_2,32_ = 4.44, p < 0.05). Post hoc analyses revealed that ΔSR improved after c-tDCS for the fast compared with the slow frequency (p < 0.01). There was a nonsignificant trend for ΔSR in the middle frequency to improve relative to the slow frequency (p = 0.058). There were no effects of frequency for ΔSR in the sham session (all p > 0.80). Furthermore, there were differences in ΔSR between c-tDCS and sham sessions for the middle (t_16_ = -1.79, p < 0.01) and fast (t_16_ = -3.38, p < 0.05) frequencies, but not the slow frequency (t_16_ = -2.31, p = 0.09). These results confirm that c-tDCS over ipsilateral M1 can reduce (i.e., improve) SR, and that the extent of improvement increases alongside the rate at which the task is performed.

Baseline SR values were predictors of ΔSR after c-tDCS (Fig. 3B) but not sham sessions (Fig. 3C), irrespective of frequency demand. The Pearson product-moment correlation coefficient showed a moderate negative association between ΔSR and baseline SR for the c-tDCS session (r = -0.40, p < 0.01) but there was no association after the sham session (r = -0.007, p = 0.95).

### c-tDCS effects on left BB MEP amplitude

**Fig 4:**
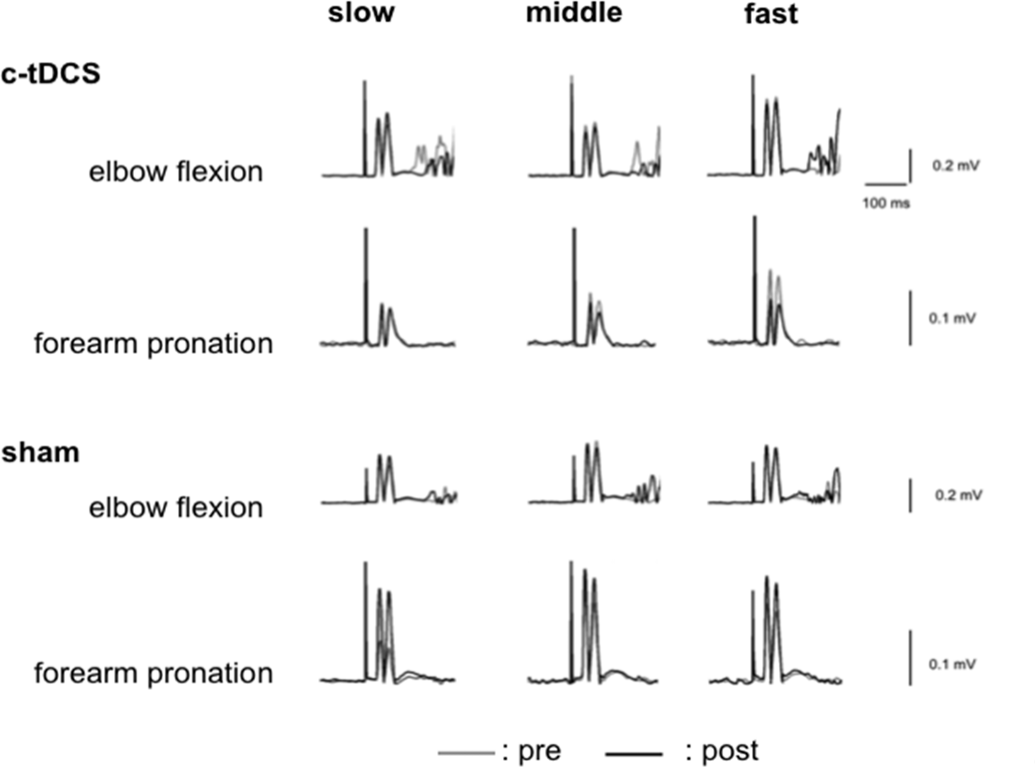
Examples of motor evoked potentials in left biceps brachii. Averaged rectified typical MEP waveforms (10 trials of each) recording from left BB prior to elbow flexion or forearm pronation in c-tDCS or sham sessions of a representative participant. The gray and black traces indicate pre and post c-tDCS or sham, respectively. Slow, middle and fast indicate 0.75, 1.0 and 1.25 Hz, respectively.

**Fig 5:**
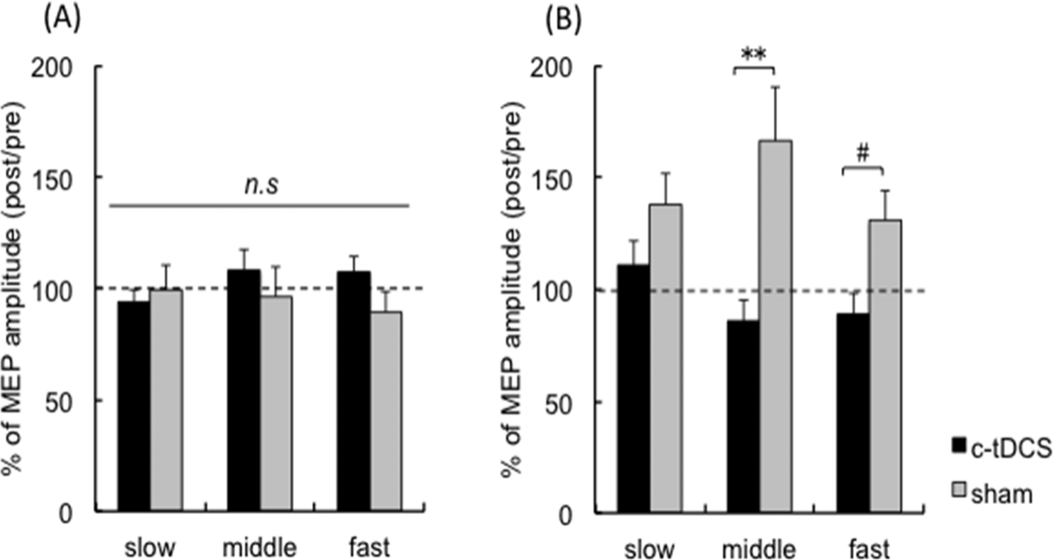
Analyses of left biceps brachi motor evoked potential amplitude. Group average BB MEP amplitude (post/pre) (*n = 17*) for the c-tDCS (filled bar) and sham (gray bar) sessions during **(A)** elbow flexion and **(B)** forearm pronation tasks. Error bars indicate SE. # p = 0.068; **p < 0.01. Slow, middle and fast indicate 0.75, 1.0 and 1.25 Hz, respectively.

Averaged rectified EMG traces with MEPs of a representative participant are shown in Fig. 4. Raw MEP amplitudes are shown in Table 1. For % change of BB MEP amplitude there was a main effect of TASK (F_1,16_ = 5.03, p <0.05), an interaction between STIM and TASK (F_1,16_ = 14.4, p <0.01) and a TASK × STIM × FREQUENCY interaction (F_2,32_ = 3.6, p <0.05), with no other effects (FREQUENCY F_2,32_ = 0.93, p = 0.41, STIM F_1,16_ = 4.18, p = 0.058, STIM × FREQUENCY F_2,32_ = 1.49, p = 0.24,TASK × FREQUENCY F_2,32_ = 0.64, p = 0.53). Post hoc tests indicated that BB MEP amplitude during elbow flexion did not differ between c-tDCS and sham (all frequencies p > 0.17) (Fig. 5A), whereas BB MEP amplitude was smaller after c-tDCS compared to sham during forearm pronation (Fig. 5B). Correction for multiple comparisons, statistical significance was retained at the middle frequency (p = 0.009), while the fast frequency indicated a nonsignificant trend (p = 0.068). BB MEP size during pronation did not differ between STIM sessions for the slow frequency (p = 0.092). These confirm a frequency dependent effect of c-tDCS on the motor system to suppress the ipsilateral BB when it is a task antagonist.

**Table 1:** Mean BB MEP amplitude (mV; ± SE) during elbow flexion and forearm pronation before and after c-tDCS and sham.

c-tDCS session					
pre			post		
slow	middle	fast	slow	middle	fast
*Elbow flexion*					
0.67 ± 0.08	0.82 ± 0.12	1.07 ± 0.14	0.64 ± 0.09	0.80 ± 0.11	1.09 ± 0.16
*Forearm pronation*					
0.16 ± 0.02	0.19 ± 0.03	0.30 ± 0.06	0.17 ± 0.03	0.15 ± 0.03	0.21 ± 0.04
**sham session**					
pre			post		
slow	middle	fast	slow	middle	fast
*Elbow flexion*					
0.47 ± 0.07	0.59 ± 0.13	0.75 ± 0.15	0.43 ± 0.06	0.44 ± 0.05	0.61 ± 0.09
*Forearm pronation*					
0.10 ± 0.01	0.11 ± 0.02	0.15 ± 0.02	0.14 ± 0.02	0.15 ± 0.02	0.18 ± 0.03

Slow, middle and fast indicate 0.75, 1.0 and 1.25 Hz, respectively.

### Control Measures

For left BB, AMT and test intensity for TMS were stable across the c-tDCS and sham sessions. AMT was 47.4 ± 11.7% and 46.0 ± 7.54% of maximum stimulator output (MSO), respectively (t_16_ = 0.71, p = 0.48). TMS intensity was 64.9 ± 8.82% and 65.0 ± 10.2% MSO, respectively (t_16_ = -0.08, p = 0.93).

The mean prestimulus rmsEMG values in left BB are summarized in Table 2. For the flexion task, there was a main effect of FREQUENCY (F_1,16_ = 25.3, p < 0.01) but no main effects of STIM (F_1,16_ = 3.94, p = 0.64), TIME (F_1,16_ = 0.23, p = 0.63) or any interactions (all F < 1, p > 0.50). For the forearm pronation task, there were no main effects of STIM (F_1,16_ = 3.53, p = 0.07, TIME (F_1,16_ = 0.49, p = 0.42), FREQUENCY (F_2,32_ = 1.47, p = 0.24) or any interactions (all others F < 1, p > 0.50). Linear regressions indicated there were no correlations between pretrigger rmsEMG and MEP amplitude (all p > 0.07).

**Table 2:** Mean prestimulus rmsEMG (μV; ± SE) in left BB muscle before and after c-tDCS and sham.

c-tDCS session					
pre			post		
slow	middle	fast	slow	middle	fast
*Elbow flexion*						
6.71 ± 0.21	6.82 ± 0.24	6.10 ± 0.23	6.66 ± 0.24	6.82 ± 0.24	7.47 ± 0.25
*Forearm pronation*						
5.92 ± 0.28	6.18 ± 0.26	6.51 ± 0.25	5.91 ± 0.02	5.93 ± 0.32	6.08 ± 0.28
**sham session**					
pre			post		
slow	middle	fast	slow	middle	fast
*Elbow flexion*					
6.10 ± 0.23	6.54 ± 0.35	6.88 ± 0.34	5.96 ± 0.21	6.28 ± 0.26	6.89 ± 0.34
*Forearm pronation*					
5.38 ± 0.18	5.52 ± 0.18	5.85 ± 0.18	5.60 ± 0.70	5.74 ± 0.27	5.86 ± 0.84

Slow, middle and fast indicate 0.75, 1.0 and 1.25 Hz, respectively.

The mean TMS-EMG burst onset interval ranged between 113 and 133 ms across the experimental sessions and is summarized in Table 3. For the elbow flexion task, there were no main effects of STIM (F_1,16_ = 0.52, p = 0.48), TIME (F_1,16_ = 3.29, p = 0.88) or FREQUENCY (F_2,32_ = 0.39, p = 0.67) and no interactions (all p > 0.055). For the forearm pronation task, there was a main effect of FREQUENCY (F_2,32_ = 5.56, p < 0.01) but no effects of STIM (F_1,16_ = 0.13, p = 0.91) or TIME (F_1,16_ = 0.15, p = 0.69) and no interactions (all p > 0.33).

**Table 3:** Mean TMS-EMG burst onset interval (ms; ± SE) during elbow flexion and forearm pronation before and after c-tDCS and sham.

c-tDCS session					
pre			post		
slow	middle	fast	slow	middle	fast
*Elbow flexion*					
114.8 ± 4.47	116.5 ± 4.73	113.4 ± 5.49	113.5 ± 5.30	114.6 ± 5.23	116.6 ± 5.57
*Forearm pronation*					
124.5 ± 6.01	127.5 ± 0.41	128.2 ± 7.34	124.6 ± 6.06	130.6 ± 6.83	132.9 ± 6.58
**sham session**					
pre			post		
slow	middle	fast	slow	middle	fast
*Elbow flexion*					
116.0 ± 6.19	117.3 ± 7.02	120.0 ± 8.04	124.3 ± 7.11	121.7 ± 7.70	125.1 ± 9.95
*Forearm pronation*					
125.5 ± 7.95	125.5 ± 8.90	130.5 ± 10.0	124.1 ± 8.48	127.2 ± 8.77	128.0 ± 8.50

Slow, middle and fast indicate 0.75, 1.0 and 1.25 Hz, respectively.

## Discussion

We designed this study to investigate whether the improvement of selective proximal upper limb muscle activation following c-tDCS depends on the frequency of muscle contraction. The findings were confirmatory and novel, and summarized as follows: 1) selective muscle activation at baseline was not dependent on frequency; 2) c-tDCS of ipsilateral M1 improved the selectivity ratio; 3) the improvement was frequency dependent; and 4) c-tDCS can selectively suppress the excitability of pathways which exert control over ipsilateral antagonist muscles.

### Effect of frequency on baseline selective muscle activation

The baseline SR values were consistent with previous studies [12,13,16,17]. Our hypothesis was that selective muscle activation, indexed by SR, would degrade as task frequency increased. This is because increased co-contraction is a feature of higher movement rates and has been described for tasks such as reaching, playing the piano, and typing on a computer keyboard [27–29]. Biomechanical analyses indicate that rapid pronation of the forearm is associated with increases in elbow joint stiffness [30]. Taken together, these studies provide evidence that muscle activation becomes less selective at higher rates of movement. However, baseline SR in the present study did not modulate as a function of the frequency of muscle contraction. One possible interpretation is that the range of frequencies employed in this study (0.75 Hz to 1.25 Hz) was too narrow. We did not test frequencies above 1.25 Hz because our neurophysiological measure of agonist-antagonist selectivity requires muscle quiescence at the time of stimulation in order for MEP amplitudes to be interpreted in a valid manner. Although we could not explore SR during very fast task rates with this paradigm, the SR results represent valid observations.

### c-tDCS and selective muscle activation

Cathodal tDCS of M1 has once again been shown to be an effective tool for suppressing neuronal excitability directed to the contralateral distal musculature (Fig. 2, c.f., [31–33]). This is strong evidence that the stimulated M1 was suppressed by c-tDCS. Therefore, any changes in the selectivity ratio (ΔSR) can be confidently attributed to brain polarization. Although it is difficult to ascertain the extent of neuronal suppression (and its spread), a conservative interpretation is that the effects on the ipsilateral hemisphere are unlikely to have been restricted to M1 and may also involve suppression of neurons within adjacent premotor cortical regions.

This study confirms earlier findings that c-tDCS of the ipsilateral M1 can improve selective muscle activation in the proximal upper limb [17], and extends these earlier results by revealing a novel frequency dependent effect. The effect was driven by BB in its role as an antagonist during forearm pronation as previously in the McCambridge et al study. Whereas BB MEPs remained unchanged before elbow flexion, across sessions, BB MEPs decreased at the middle and fast tempos for the forearm pronation task.

What accounts for this frequency-dependent effect? It is possible that the ipsilateral M1 activity as well as recruitment of ipsilateral descending motor pathways projecting to propriospinal neurons might differ depending upon frequency demands of muscle contraction [34]. In TMS studies, changes in ipsilateral M1 excitability have been noted when performing repetitive rhythmic muscle contraction under varying frequency demands [20,21]. Functional magnetic resonance imaging has revealed ipsilateral M1 deactivation at low (i.e., 0.25Hz) compared to fast frequencies (i.e., up to 4Hz), and ipsilateral M1 activation scaled linearly with movement frequency [35]. Further, the recruited brain networks may differ between discrete (i.e., relatively slow) and rhythmic (i.e., relatively fast) manual movements [36]. In our task the slow tempo may not have increased ipsilateral M1 excitability thus limiting the effects of c-tDCS of ipsilateral M1 to the higher movement rates.

Perhaps surprisingly, ΔSR worsened over the course of the sham session. One possible interpretation is that the task may have induced fatigue as participants performed 900 muscle contractions in each session while keeping pace at the prescribed tempo. Although this idea was not tested directly, it is worth noting that MEP size tends to increase in fatigued muscle performing a sustained muscle contraction at a high muscle force output level [37,38]. Given that BB MEP tended to increase in the forearm pronation task with sham stimulation, and this effect was attenuated with c-tDCS, it may indicate that the effects can be even greater in the presence of fatigue. This would require further investigation, as might other possibilities for the observed effect. In summary, there is a possibility that c-tDCS of the ipsilateral M1 might attenuate the worsening of SR between pre and post c-tDCS.

Our study did not examine interhemispheric interactions or their contribution to selective muscle activation in the proximal upper limb. There is some evidence that the degree of interhemispheric inhibition (IHI), is greater for distal muscles than BB or triceps brachii muscles [39]. Previously it was shown that ipsilateral silent periods, a measure that reflects at least in part, interhemipsheric inhibition was unchanged by c-tDCS of the ipsilateral M1 [15,17]. The previous findings along with the current results suggest that improvements of selective muscle activation after c-tDCS are due to effects on uncrossed ipsilateral pathways, as opposed to interhemispheric cortical mechanisms.

## Conclusions

We provide novel evidence that c-tDCS is an effective tool for improving selective muscle activation. Namely, its efficacy depends on the frequency demands of the imposed task, and is greater when frequency demands are presumably high enough to recruit activation in the ipsilateral M1. After stroke which results in upper limb impairment, some patients exhibit abnormal synergies that worsen with increasing movement speed [40]. Given the speed-dependent effects on the expression of abnormal muscle synergies, c-tDCS of the contralesional M1 may be applicable in rehabilitation settings for improving movements of the paretic upper limb.

## Supporting Information

S1 Data This is the supporting information file. (ZIP)

## References

[1] Marsden CD, Obeso JA, Rothwell JC. The function of the antagonist muscle during fast limb movements in man. J Physiol. 1983; 335: 1–13. 6875870 10.1113/jphysiol.1983.sp014514 PMC1197333

[2] Wierzbicka MM, Wiegner AW, Shahani BT. Role of agonist and antagonist muscles in fast arm movements in man. Exp Brain Res. 1986; 63: 331–340. 3758250 10.1007/BF00236850

[3] De Luca CJ, Mambrito B. Voluntary control of motor units in human antagonist muscles: coactivation and reciprocal activation. J Neurophysiol. 1987; 58: 525–542. 3655881 10.1152/jn.1987.58.3.525

[4] Cheney PD, Fetz EE, Palmer SS. Patterns of facilitation and suppression of antagonist forelimb muscles from motor cortex sites in the awake monkey. J Neurophysiol. 1985; 53: 805–820. 2984355 10.1152/jn.1985.53.3.805

[5] Bertolasi L, Priori A, Tinazzi M, Bertasi V, Rothwell JC. Inhibitory action of forearm flexor muscle afferents on corticospinal outputs to antagonist muscles in humans. J Physiol. 1998; 511: 947–956. 9714872 10.1111/j.1469-7793.1998.947bg.x PMC2231145

[6] Hortobágyi T, del Olmo MF, Rothwell JC. Age reduces cortical reciprocal inhibition in humans. Exp Brain Res. 2006; 171: 322–329. 16307241 10.1007/s00221-005-0274-9

[7] Brunnstrom S. Movement Therapy in Hemiplegia: a Neurophysiological Approach. New York: Harper & Row; 1970

[8] Levin MF, Selles RW, Verheul MH., Meijer OG. Deficits in the coordination of agonist and antagonist muscles in stroke patients: implications for normal motor control. Brain Res. 2000; 853: 352–369. 10640634 10.1016/s0006-8993(99)02298-2

[9] Gowland C, DeBruin H, Basmajian J V, Plews N, Burcea I. Agonist and antagonist activity during voluntary upper-limb movement in patients with stroke. Phys Ther. 1992; 72: 624–633. 1508970 10.1093/ptj/72.9.624

[10] Welmer A, Holmqvist L, Sommerfeld D. Hemiplegic limb synergeis in stroke patients. Am J Phys Med Rehabil. 2006; 85: 112–119. 16428901 10.1097/01.phm.0000197587.78140.17

[11] Gerachshenko T, Rymer W, Stinear J. Abnormal corticomotor excitability assessed in biceps brachii preceding pronator contraction post-stroke. Clin Neurophysiol. 2008; 119: 683–692. 10.1016/j.clinph.2007.11.004 18164237 PMC2288665

[12] Bradnam LV, Stinear CM, Barber PA, Byblow WD. Contralesional hemisphere control of the proximal paretic upper limb following stroke. Cereb cortex 2012; 22: 2662–2671. 10.1093/cercor/bhr344 22139791 PMC4705341

[13] Gerachshenko T, Stinear J. Suppression of motor evoked potentials in biceps brachii preceding pronator contraction. Exp Brain Res. 2007; 183: 531–539. 17665175 10.1007/s00221-007-1071-4

[14] Bradnam L, Stinear C, Lewis G, Byblow W. Task-dependent modulation of inputs to proximal upper limb following transcranial direct current stimulation of primary motor cortex. J Neurophysiol. 2010; 103: 2382–2389. 10.1152/jn.01046.2009 20220073

[15] Bradnam LV, Stinear CM, Byblow WD. Cathodal transcranial direct current stimulation suppresses ipsilateral projections to presumed propriospinal neurons of the proximal upper limb. J Neurophysiol. 2011; 105: 2582–2589. 10.1152/jn.01084.2010 21389299

[16] Bradnam LV, Stinear CM, Byblow WD. Theta burst stimulation of human primary motor cortex degrades selective muscle activation in the ipsilateral arm. J Neurophysiol. 2010; 104: 2594–2602. 10.1152/jn.00365.2010 20844109

[17] McCambridge AB, Bradnam L V, Stinear CM, Byblow WD. Cathodal transcranial direct current stimulation of the primary motor cortex improves selective muscle activation in the ipsilateral arm. J Neurophysiol. 2011; 105: 2937–2942. 10.1152/jn.00171.2011 21511707

[18] Van den Berg FE, Swinnen SP, Wenderoth N. Involvement of the primary motor cortex in controlling movements executed with the ipsilateral hand differs between left- and right-handers. J Cogn Neurosci. 2011; 23: 3456–3469. 10.1162/jocn_a_00018 21452954

[19] Verstynen T, Diedrichsen J, Albert N, Aparicio P, Ivry RB. Ipsilateral motor cortex activity during unimanual hand movements relates to task complexity. J Neurophysiol. 2005; 93: 1209–1222. 15525809 10.1152/jn.00720.2004

[20] Uehara K, Morishita T, Funase K. Excitability changes in the ipsilateral primary motor cortex during rhythmic contraction of finger muscles. Neurosci Lett. 2011; 488: 22–25. 10.1016/j.neulet.2010.10.073 21056628

[21] Uehara K, Morishita T, Kubota S, Funase K. Neural mechanisms underlying the changes in ipsilateral primary motor cortex excitability during unilateral rhythmic muscle contraction. Behav Brain Res. 2013; 240: 33–45. 10.1016/j.bbr.2012.10.053 23174210

[22] Oldfield RC. The assessment and analysis of handedness: The Edinburgh inventory. Neuropsychologia 1971; 9: 97–113. 5146491 10.1016/0028-3932(71)90067-4

[23] Kaneko K, Kawai S, Fuchigami Y, Morita H, Ofuji A. The effect of current direction induced by transcranial magnetic stimulation on the corticospinal excitability in human brain. Electroencephalogr Clin Neurophysiol. 1996; 101: 478–482. 9020819 10.1016/s0013-4694(96)96021-x

[24] Nakamura H, Kitagawa H, Kawaguchi Y, Tsuji H. Intracortical facilitation and inhibition after transcranial magnetic stimulation in conscious humans. J Physiol. 1997; 498: 817–823. 9051592 10.1113/jphysiol.1997.sp021905 PMC1159197

[25] Nitsche MA, Liebetanz D, Lang N, Antal A, Tergau F, Paulus W. Safety criteria for transcranial direct current stimulation (tDCS) in humans. Clin Neurophysiol. 2003; 114: 2220–2222. 14580622 10.1016/s1388-2457(03)00235-9

[26] Rom DM. A sequentially rejective test procedure based on a modified Bonferroni inequality. Biometrika 1990; 77: 663–665.

[27] D’Avella A, Fernandez L, Portone A, Lacquaniti F. Modulation of phasic and tonic muscle synergies with reaching direction and speed. J Neurophysiol. 2008; 100: 1433–1454. 10.1152/jn.01377.2007 18596190

[28] Furuya S, Aoki T, Nakahara H, Kinoshita H. Individual differences in the biomechanical effect of loudness and tempo on upper-limb movements during repetitive piano keystrokes. Hum Mov Sci. 2012; 31: 26–39. 10.1016/j.humov.2011.01.002 21816497

[29] Gerard MJ, Armstrong TJ, Martin BJ, Rempel DA. The Effects of Work Pace on Within-Participant and Between-Participant Keying Force, Electromyography, and Fatigue. Hum Factors J Hum Factors Ergon Soc. 2002; 44: 51–61. 10.1518/0018720024494757 12118873

[30] Kuxhaus L, Zeng S, Robinson CJ. Dependence of elbow joint stiffness measurements on speed, angle, and muscle contraction level. J Biomech. 2014; 47: 1234–1237. 10.1016/j.jbiomech.2013.12.008 24433667

[31] Nitsche M, Paulus W. Excitability changes induced in the human motor cortex by weak transcranial direct current stimulation. J Physiol. 2000; 527: 633–639. 10990547 10.1111/j.1469-7793.2000.t01-1-00633.x PMC2270099

[32] Nitsche M, Nitsche M, Klein C. Level of action of cathodal DC polarisation induced inhibition of the human motor cortex. Clin Neurophysiol. 2003; 114: 600–604. 12686268 10.1016/s1388-2457(02)00412-1

[33] Monte-Silva K, Kuo M, Liebetanz D, Paulus W, Nitsche M. Shaping the optimal repetition interval for cathodal transcranial direct current stimulation (tDCS). J Neurophysiol. 2010; 27: 1735–1740. 10.1152/jn.00924.2009 20107115

[34] Bradnam L V, Stinear CM, Byblow WD. Ipsilateral motor pathways after stroke: implications for non-invasive brain stimulation. Front Hum Neurosci. 2013; 7: 184 10.3389/fnhum.2013.00184 23658541 PMC3647244

[35] Hayashi MJ, Saito DN, Aramaki Y, Asai T, Fujibayashi Y, Sadato N. Hemispheric Asymmetry of frequency- dependent suppression in the ipsilateral primary motor cortex during finger movement: a functional magnetic resonance imaging study. Cereb cortex 2008; 18: 2932–2940. 10.1093/cercor/bhn053 18413350 PMC2583153

[36] Schaal S, Sternad D, Osu R, Kawato M. Rhythmic arm movement is not discrete. Nat Neurosci. 2004; 7: 1137–1145. 10.1038/nn1322 15452580

[37] Taylor JL, Gandevia SC. Transcranial magnetic stimulation and human muscle fatigue. Muscle Nerve 2001; 24: 18–29. 11150962 10.1002/1097-4598(200101)24:1<18::aid-mus2>3.0.co;2-d

[38] Taylor J, Butler J. Changes in motor cortical excitability during human muscle fatigue. J Physiol. 1996; 490: 519–528. 8821148 10.1113/jphysiol.1996.sp021163 PMC1158688

[39] Harris-Love ML, Perez MA, Chen R, Cohen LG. Interhemispheric inhibition in distal and proximal arm representations in the primary motor cortex. J Neurophysiol. 2007; 97: 2511–2515. 17215494 10.1152/jn.01331.2006

[40] Simkins M, Burleigh A, Jacob J. Rhythmic affects on stroke—induced joint synergies across a range of speeds. Exp brain Res. 2013; 229: 517–524. 10.1007/s00221-013-3613-2 23793525

